# Diabetic complications and prospective immunotherapy

**DOI:** 10.3389/fimmu.2023.1219598

**Published:** 2023-07-07

**Authors:** Lewis Reynolds, Zhengkang Luo, Kailash Singh

**Affiliations:** Department of Medical Cell Biology, Uppsala University, Uppsala, Sweden

**Keywords:** diabetes mellitus, type 1 diabetes (T1D), type 2 diabetes (T2D), diabetes complications, immunotherapy, inflammation, regulatory B (Breg) cells, regulatory T (Treg) cells

## Abstract

The incidence of Diabetes Mellitus is increasing globally. Individuals who have been burdened with diabetes for many years often develop complications as a result of hyperglycemia. More and more research is being conducted highlighting inflammation as an important factor in disease progression. In all kinds of diabetes, hyperglycemia leads to activation of alternative glucose metabolic pathways, resulting in problematic by-products including reactive oxygen species and advanced glycation end products. This review takes a look into the pathogenesis of three specific diabetic complications; retinopathy, nephropathy and neuropathy as well as their current treatment options. By considering recent research papers investigating the effects of immunotherapy on relevant conditions in animal models, multiple strategies are suggested for future treatment and prevention of diabetic complications with an emphasis on molecular targets associated with the inflammation.

## Introduction

Diabetes Mellitus is a chronic metabolic disease causing those with the condition to be subjected to higher blood glucose ranges compared to healthy controls. According to the World Health Organization (WHO) an estimated 422 million people worldwide live with diabetes and 1.5 million global deaths are attributed to complications from the disease (1). Diabetic incidence worldwide has shown an increase over time, with a predicted 578 million cases by 2030 and a predicted total of 700 million by 2045 (2).

Hyperglycemia seen in diabetics stems from either the inability to produce insulin through beta cell loss (Type 1 Diabetes, T1D) or through an accumulated resistance to insulin (Type 2 Diabetes, T2D). Despite the difference in disease causation, chronic hyperglycemia remains problematic for all types of diabetes. Current recommendations for diabetics, in addition to increased control of their glycemic range *via* insulin injection or insulin sensitivity medication, is to eat a healthy balanced diet in addition to exercise in order to reduce the risk of long-term complications (3).

**Figure 1:**
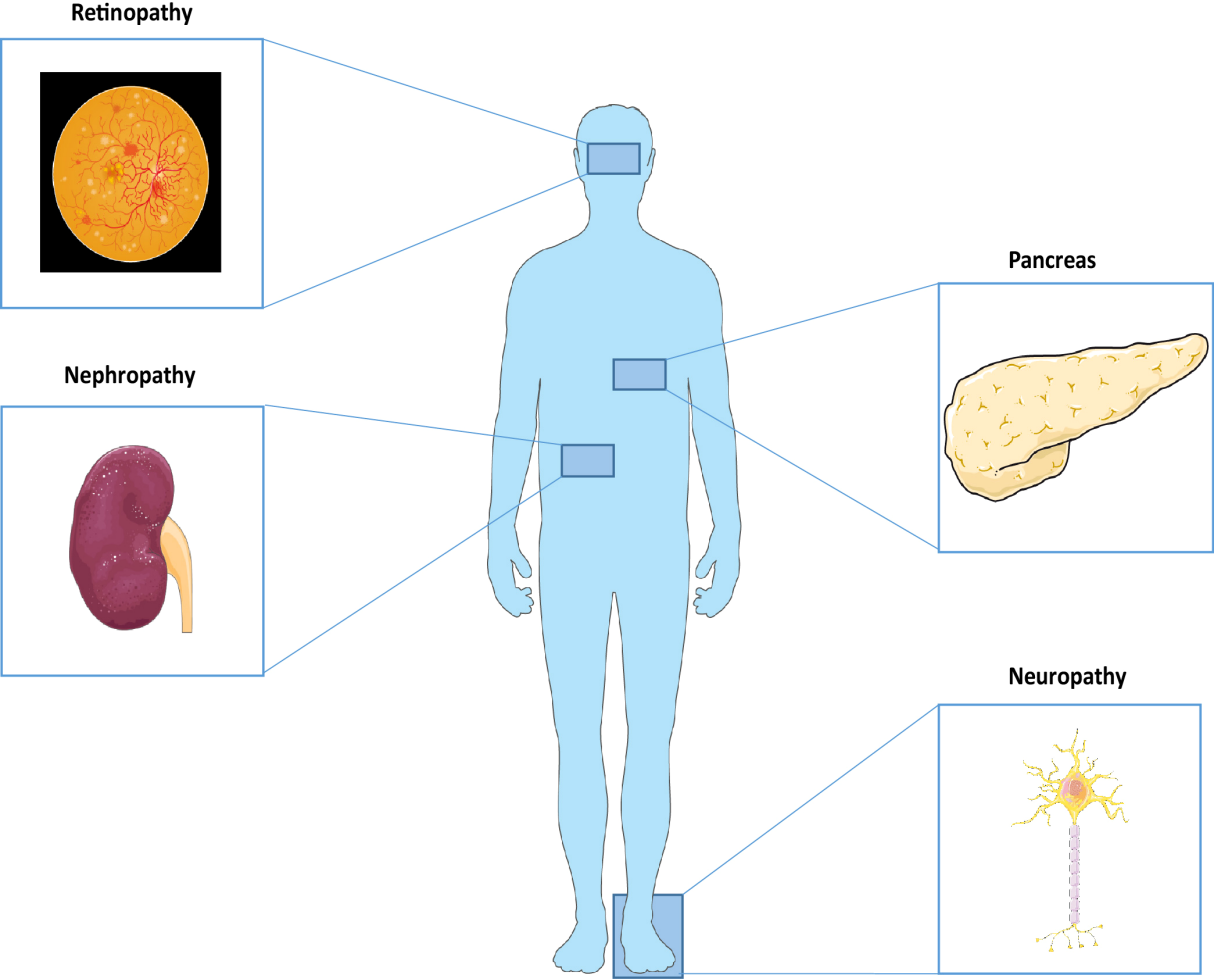
Microvascular damage affects the eyes, kidneys and nerves in diabetes. The three major organs affected by microvascular complications due to an extended exposure to hyperglycemic conditions. Parts of the figure were drawn by using pictures from Servier Medical Art. Servier Medical Art by Servier is licensed under a Creative Commons Attribution 3.0 Unported License (https://creativecommons.org/licenses/by/3.0/).

Complications from diabetes can generally be categorized into two main groups. Damage to large arteries can lead to problems with the heart, brain and legs and are often referred to as macrovascular complications. Microvascular complications, by contrast, affect the smaller blood vessels in many organs throughout the body, including the eyes, kidneys and nerves referred to as retinopathy, nephropathy and neuropathy, respectively (**Figure 1**).

With a global increase in the number of diabetics each year, and a large enough percentage of diabetics statistically likely to develop complications as a result of diabetes, the quality of life and cost for individuals/health care institutions are sure to be negatively affected. With as many as 1 in 9 adults dying as a result of their diabetes, the prevention of diabetes and its complications is very important, especially in middle income countries (4). Currently medication is given to treat symptoms of diabetic complications without targeting the underlying cause of the disease progression. This analysis aims to review the current literary understanding of the role of the immune system in diabetic complications namely; retinopathy, nephropathy and neuropathy, and suggest treatment based on the advancements made in immunotherapy.

## Microvascular complications of diabetes

### Diabetic retinopathy

Diabetic Retinopathy (DR) is the most common complication to affect individuals with diabetes (5, 6). As of 2022, the number of diabetics with DR stands at around 93 million cases globally. Prevalence of DR is as much as 77.3% in T1D and 25.1% in T2D. Approximately 25-30% of these cases are thought to progress to vision threatening diabetic macular edema, 5-8% of which will require laser surgery to correct and another 5% requiring vitrectomy surgery (7–9). Although not life threatening, DR may lead to microvascular damage to the retina resulting in blindness (7, 9).

In response to hyperglycemia, alternative pathways of glucose metabolism are activated ultimately leading to cellular damage over a sustained amount of time (10–12). The polyol pathway is one such pathway in which glucose is reduced to sorbitol and then converted to fructose in the presence of sorbitol dehydrogenase (13). Sorbitol dehydrogenase is present in most tissues; however, it is absent in key tissues associated with diabetic complications, namely the eyes, kidneys and nerves (14, 15). Müller cells of the eyes as well as the retinal ganglia, vascular pericytes and endothelial cells do however contain the enzyme aldose reductase (16–19), responsible for the initial conversion of glucose to sorbitol. The excess of sorbitol leads to osmotic pressures in the retina due to its impermeability, leading to edema. The pathway further produces reactive oxygen species (ROS), advanced glycation end products (AGEs), as well as protein kinase C activation further contributing to disease progression (7, 20).

The presence or absence of neovascularization is used to categorize DR into one of two categories - proliferative or non-proliferative, respectively. Proliferative is the most advanced form of the disease, where hypoxia as a result of decreased vascular function by capillary occlusion drives neovascularization (21–26). Loss of vision caused by macular edema is common in both forms of DR, however, blindness caused by bleeding, hemorrhage and subsequent retinal detachment are benchmarks of proliferative DR (27, 28).

**Figure 2:**
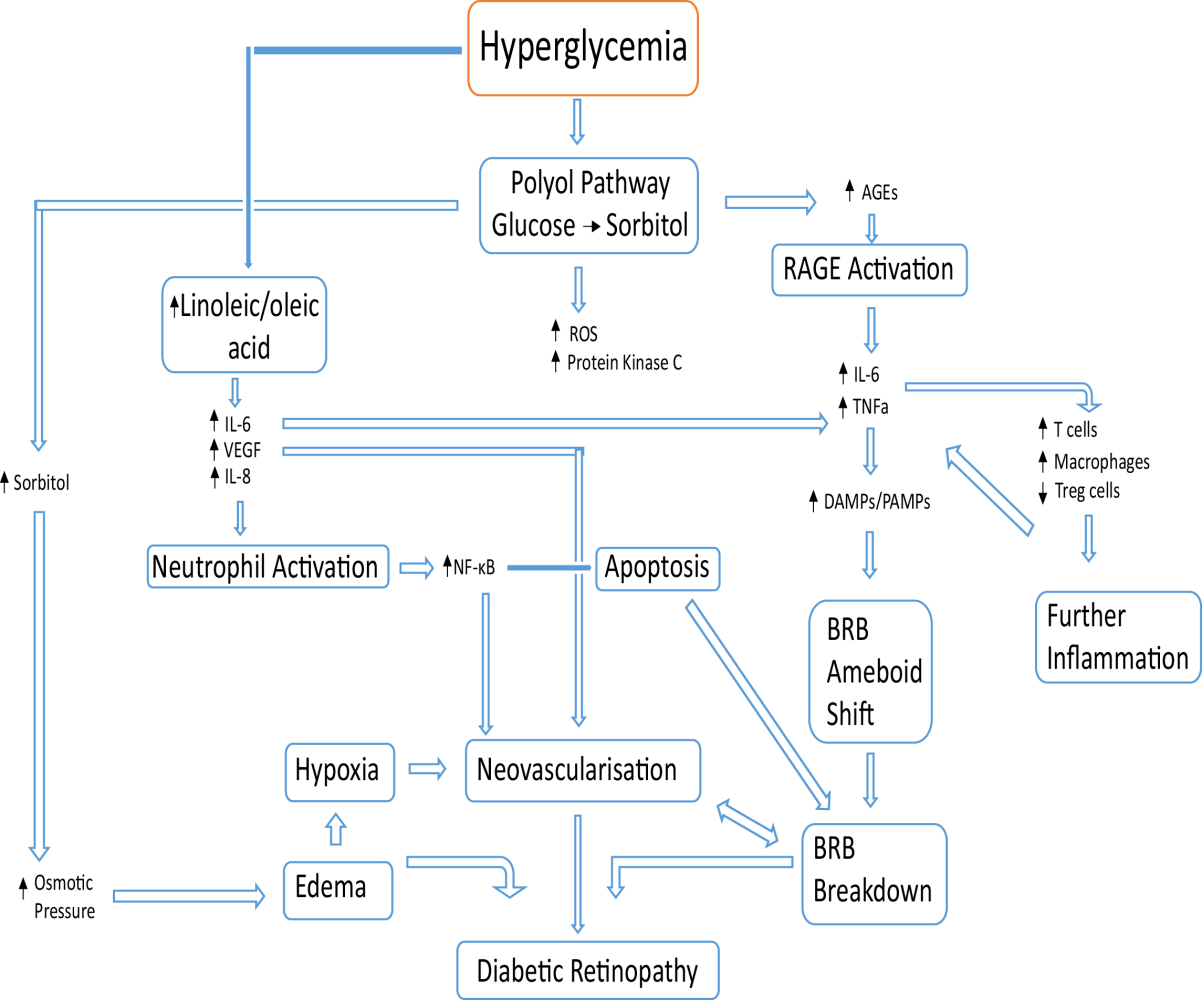
Basic overview of pathological events leading to DR. Beginning with hyperglycemia, alternative glucose metabolic pathways create excess sorbitol and cytotoxic end products. The increase in advanced glycation end products results in more binding with the corresponding receptor (RAGE) causing an increase in proinflammatory cytokines (IL-6, IL-8 and VEGF). An increase in linoleic and oleic acid increases the levels of IL-6, IL-8 and VEGF, which activate neutrophils. The blood retinal barrier breakdown is caused by apoptosis from neutrophil activation in conjunction with damage-associated molecular patterns (DAMPs) and pathogen-associated molecular patterns (PAMPs) activation causing a Blood Retinal Barrier (BRB) phenotype shift to a more ameboid state. DR results from BRB breakdown, local neovascularization and edema.

The presence of neovascularization and edema in DR provides evidence of a defining hallmark of inflammation (21, 29, 30). DR was not considered an inflammatory disease until recently, as the retina was originally considered an immune privileged tissue. This is true at default, where no inflammatory markers should be present. However, the increase in proinflammatory markers during diabetic retinopathy highlights the important inflammatory shift associated with the condition (31). In an experiment by Capozzi et al., concentrations of Interleukin-6 (IL-6), IL-8 and vascular endothelial growth factor (VEGF) were significantly increased by human Müller cells in the presence of both linoleic and oleic acid (32). When treated with D or L-glucose alone, an increase in the production of VEGF was observed in Müller cells. This was attributed by the group to the osmotic effects of increased blood glucose. Dietary levels of polyunsaturated fatty acids (like linoleic acid) have been shown to correlate with the incidence of retinopathy in T2D and has been suggested as a biomarker in T2 diabetes (33). These fatty acids collect in Müller cells, spanning the retina leading to inflammation from the increase of proinflammatory cytokines IL-6 and IL-8 among other factors (32, 34–38) (**Figure 2**).

Increased levels of IL-8 result in neutrophil activation at the activated epithelium (32, 39). Activation of the neutrophil results in extracellular trap formation which further contributes to the pathogenesis of DR (39, 40). In addition to this, IL-8 is associated with the activation of the nuclear factor κB (NF-κB) pathway, which is present in many cell types and participates in cell apoptosis and neovascularization (41). Over time, an accumulation of NF-κB expression due to hypoxia, hyperglycemia, AGEs and some inflammatory cytokines, triggers an increase in VEGF expression and thus, neovascularization (41). The apoptotic breakdown of the blood-retinal barrier (BRB), either by endothelial cell death or supporting cell death (including Müller cells), damages the BRB microenvironment causing leakage of blood and negatively affects proper retinal barrier function (42) (**Figure 2**).

Before the degradation of the blood-retina barrier, the only immune sentinels are the microglia (43). However, upon the integrity loss of the blood-retina barrier, products of hyperglycemia may alter the physiology of microglia to initiate inflammation, for example AGEs binding with their RAGE receptor (receptor for advanced glycation end products) on microglia, resulting in secretion of proinflammatory IL-6 and TNFα (44). This BRB degradation is achieved by activation of the receptors of damage-associated molecular patterns (DAMPs) and pathogen-associated molecular patterns (PAMPs) located on the microglia, causing them to divide and shift morphology to a more amoeboid nature (31, 44) as seen in **Figure 2**.

Tumor necrosis factor alpha (TNFα) is a proinflammatory cytokine commonly produced by both macrophages and T cells which is responsible for inducing inflammation and apoptosis (45). In DR, TNFα appears to actively participate in the pathogenesis of inflammatory, edematous, neovascular, and neurodegenerative diseases (45, 46). In studies examining concentrations of TNFα in the vitreous, TNFα appears early and concentrations increase throughout disease progression (31, 47). TNFα and Interleukin-1 beta (IL-1β) have been shown by *in vitro* studies to cause microglia and endothelial cells to secrete VEGF (31, 47). IL-1β has also been noted to feature in cross-talk with IL-8, where IL-1β’s presence has initiated the release of IL-8, causing more inflammation *in vivo* in both human and rodent models (31, 48).

Apart from surgical options (including laser treatment), currently prescribed medication includes anti-VEGF agents (monoclonal antibodies, mABs) and steroid injections (49). VEGF secreting Müller cells have been shown to break down the blood-retina barrier and cause neovascularization in rodent models of DR (50). Anti-VEGF mABs (such as; bevacizumab, ranibizumab, and aflibercept (50)) may be administered as treatment, with Ranibizumab approved intravitreal use in Europe and the US. Intravitreal injection has seen some good results, however these injections must be taken continuously to maintain the therapeutic effect (51). An article by Nair and Modi proposed that anti-VEGF agents may in fact be masking DR’s true stage rather than treating the underlying pathology (52).

Medication targeting the influence of the immune system would in theory decrease the development of DR. TNFα blockers are already used in other disease treatments, however they are relatively new and long-term use still needs to be evaluated. Monoclonal antibodies also exert some positive effects on DR, where trials using combinations increase therapeutic effects (53). Microglia can form proinflammatory M1 phenotypes (CD26 and CD32 markers), as well as an anti-inflammatory M2 phenotype (CD163 and CD206 markers) where anti-inflammatory molecules such as IL-4, IL-10, IL-13 and transforming growth factor beta (TGFβ) are excreted (44, 54, 55). In the presence of chronic disease and danger signals (seen in chronic hyperglycemia) the phenotypic shift of microglia from balanced to proinflammatory reacts as a positive feedback mechanism, which can further disease progression (44). By taking advantage of the cellular communication environment, namely cytokines and receptors, research into the M1 to M2 phenotype shift is possible. By shifting the balance to an anti-inflammatory M2 phenotype, the homeostasis and protective effects should return in theory. Research has been conducted on M1 to M2 phenotype shift in neurodegenerative disorders (55), however more investigation is needed in DR.

### Diabetic nephropathy

Diabetic kidney disease (DKD) can affect as many as 30% T1D patients and 40% of T2D patients, making diabetes accountable for 20-50% of those entering renal failure programs (56, 57). It is the leading contributor of End Stage Renal Disease (ESRD) in the US, prompting dialysis and kidney transplantation (58, 59). Of these ESRD cases, it is observed that 80% are due to diabetes or hypertension, with a 5-year survival rate of less than 40% (60). Diabetic Nephropathy (DN) is an umbrella term used for kidney diseases associated with the above conditions in diabetics. It presents as a chronic inflammation of the kidney(s) alongside proteinuria (albuminuria) and decline in renal functioning (61).

DN progresses in stages using different pathological benchmarks. In early stages of the disease, fibrosis is caused by glomerular and tubular hypertrophy ultimately caused by hyperglycemia, as well as a multitude of other hemodynamic factors (58, 62). This causes a thickening of the basement membrane and an expansion of the mesangium in later stages, leading to end-stage glomerular closure and tubulointerstitial fibrosis as well as cellular stress (63–68). The advanced stage of disease can be characterized by an infiltration of immune cells to the damaged kidney environment (61, 69). Disease progression usually occurs over 10-20 years, however, this can vary for different patients depending on glycemic control among other factors (58).

**Figure 3:**
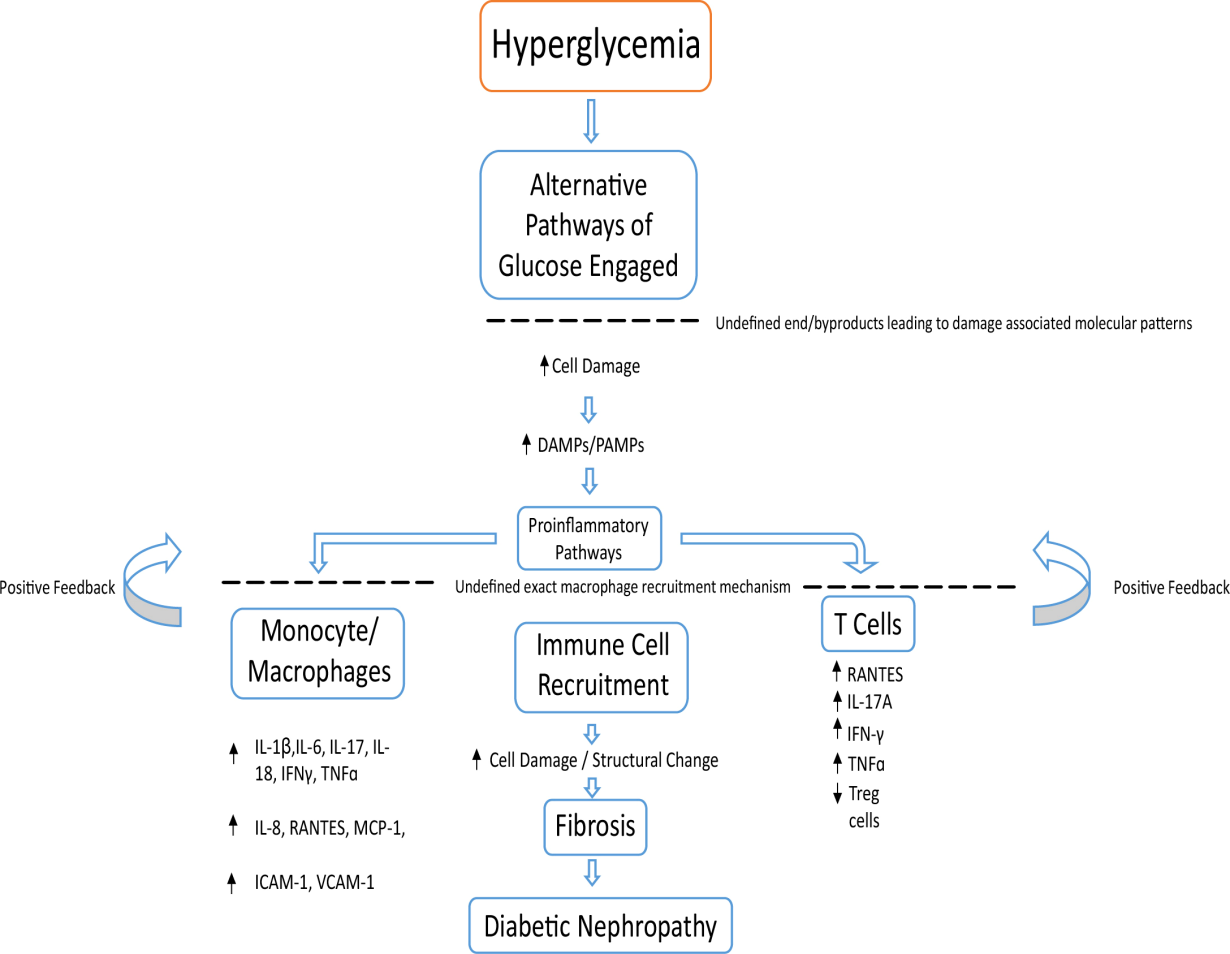
Basic overview of possible pathological events leading to DN. Chronic hyperglycemia leading to cellular damage increases DAMPs and PAMPs. This encourages inflammatory cells such as monocytes, macrophages, T cells and neutrophils to begin a positive feedback loop of immune cell recruitment and inflammation. This leads to kidney fibrosis and a decline in kidney function, leading to DN.

The pathobiology of the disease is a lot more complex in reality, with changes to kidney structure, function and metabolism that still remain to be better understood (70). Hyperglycemia leading to alternatively activated glucose pathways and their cytotoxic products are just one of many associated causes of disease onset, with other factors such as immune response, oxidative stress and oxidized lipids also playing distinct roles. All of these combined cause damage to the kidney cells, releasing DAMPs which trigger proinflammatory pathways (61) (**Figure 3**). The amount of proinflammatory cytokines both circulating and synthesized by the kidney increase during DKD (71–73).

The recruitment of monocytes and macrophages (74, 75) responding to damaged cells of the renal mesangium, podocytes and endothelial cells causes surrounding cells to release various proinflammatory cytokines (IL-1β, IL-6, IL-8, IL-17, IL-18, interferon gamma (IFNγ), TNFɑ), chemokines (RANTES, and monocyte chemoattractant protein-1 (MCP-1); regulated on activation, normal T cell expressed and secreted (76) adhesion molecules; intracellular adhesion molecule-1 (ICAM-1) and vascular cell adhesion molecule-1 (VCAM-1), begetting a vicious cycle of chronic inflammation. This results in an accumulation of immune cells from both the innate and adaptive systems (77). Such inflammation drives structural changes in DN through fibrosis (63, 75) (**Figure 3**). The amount of proinflammatory cytokines both circulating and synthesized by the kidney increase during DKD (71–73). This is seen in urine samples and renal biopsies of patients with DKD, where an increase of IL-6 and IL-8 compared to healthy controls is observed (71, 78).

F4/80 or CD68 positive macrophages are often found in kidneys of DN patients *via* flow cytometry and immunohistochemical staining (61, 79). Depletion of macrophages conducted by You et al. showed that by removing macrophages in streptozotocin (STZ) model mice, one can better protect renal tissue. You et al. were the first to show evidence for direct interaction between podocytes and M1 macrophages, with impairment of podocyte integrity potentially due to the action of MCP-1 (80). As it stands, the precise nature of how macrophages are recruited to the kidneys remains to be clarified. Cell adhesion molecules and chemokines/chemokine receptors have been found to be involved in this process with the vascular endothelium overexpressing cell adhesion molecules on its surface, including ICAM-1 and VCAM-1, which recruit precursor macrophages (61). T cells accompany macrophages to the kidney and progress DN through the secretion of proinflammatory cytokines such as TNFɑ and INFɣ (61, 63, 75, 81, 82). A study published by Moon et al. also demonstrated that activated T cells were attributed to aberrant diabetic kidney injury in STZ mouse models in conjunction with hyperglycemia (83). As with macrophages, the mechanism of T cell recruitment to the kidneys remains unclarified, although as before it is known to involve proinflammatory cytokines, chemokines and adhesion molecules (75).

T cells, much like other immune cells, feature a great deal of plasticity (84–88). They can alter their phenotype in order to become the subset required in a given environment based on extracellular clues (85). It has been well documented that T cells have many subsets, including Th1, Th2, Th17 and Treg cells (89, 90). T cells which are CD4^+^ have been shown to interact with fibroblasts to induce fibrosis (91). In a study by Peng et al., the expression levels of IL-17A by renal T cell subsets was monitored in obstructed kidneys (91). It was found that Th17 T cells, γδ T lymphocytes and a population of CD3^+^CD4^−^CD8^−^γδTCR^−^ cells produced IL-17A after obstructive injury in the kidney. They further demonstrated using IL-17A deficient mice that IL-17A enhanced the production of RANTES, a proinflammatory chemokine involved in the recruitment of T cells and monocytes leading to fibrosis. Th17 cells are known to secrete the most IL-17A (91). Their phenotype is induced by cytokines TGFβ and IL-6, levels of which increase during obstructive kidney injury (91–93).

In the past two decades, no new medication has been suggested in order to restore kidney function or prevent the further loss of kidney function (94–96). Currently, treatment of DN is blood glucose/blood pressure control, lipid lowering and renin-angiotensin system blockade. A major issue with these therapies however is that they are generally prescribed at the later stage of disease development, offering only some reno-protective element (70). Drug delivery to injured kidneys also requires high dosage, often associated with adverse effects (61). Given the above evidence for the role of the immune system in DN, consideration must be given to including immunotherapy as a means to treat DN and slow/stop disease progression.

### Diabetic neuropathy

Diabetic Neuropathy is a term used to encapsulate the many clinical and sub-clinical issues affecting a broad range of tissues *via* different underlying molecular mechanisms (97). Clinical manifestations of diabetic neuropathy may be grouped under either diffuse or focal neuropathies (97), where diffuse tends to affect many nerves connected to distal parts of the body such as the feet. Diabetic Peripheral Neuropathy (DPN) is the most common subtype of the peripheral neuropathies (98). Approximately 50% of adults with diabetes will develop DPN at some stage in their life (99). Of these 50%, foot ulceration is common, with some patients even requiring foot amputation later on (100, 101). Diabetes can produce several types of peripheral nervous damage, the most common being bilateral and symmetric damage of the feet (102). Although the foot is the most common area affected by complications of the nervous system, diabetic neuropathies have the potential to affect many organs thereby lowering the quality of life and increasing morbidity (103).

**Figure 4:**
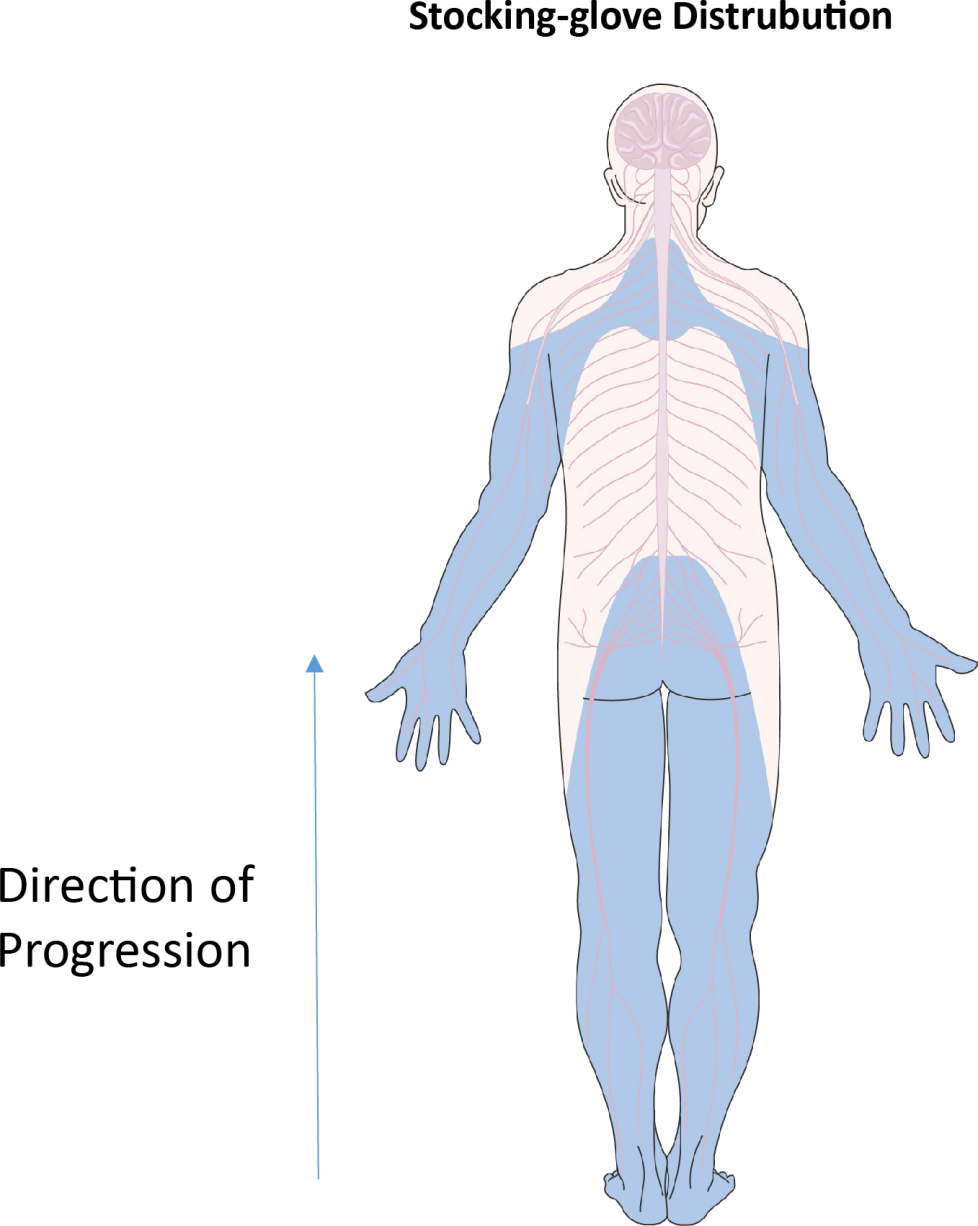
“Stocking-glove” distribution. Blue areas indicate regions affected by nerve damage in diabetic neuropathy. Beginning in the feet, nerve damage rises through the legs. Similar events happen in the arms, beginning in the hands and moving upwards in a distal to proximal direction. Parts of the figure were drawn by using pictures from Servier Medical Art. Servier Medical Art by Servier is licensed under a Creative Commons Attribution 3.0 Unported License (https://creativecommons.org/licenses/by/3.0/).

The peripheral nervous system (PNS) is more exposed to environmental factors compared to the central nervous system. The types of nerves in the PNS range from highly myelinated motor nerves, less myelinated sensory nerves and lightly myelinated autonomic nerves. This is often dictated by length, where motor neurons tend to be longer and therefore more myelinated compared to sensory nerves for pain and temperature sensations (104). Symptoms of diabetic neuropathy usually start with a lack of sensation, over time developing further to more positive symptoms such as burning sensations, pins and needles, tingling and/or lightning pain (97). As DPN progresses, demyelination, beginning in the feet, moves up the legs and eventually reaches the hands in what is known as the “stocking-glove” distribution (105) as demonstrated in **Figure 4**.

**Figure 5:**
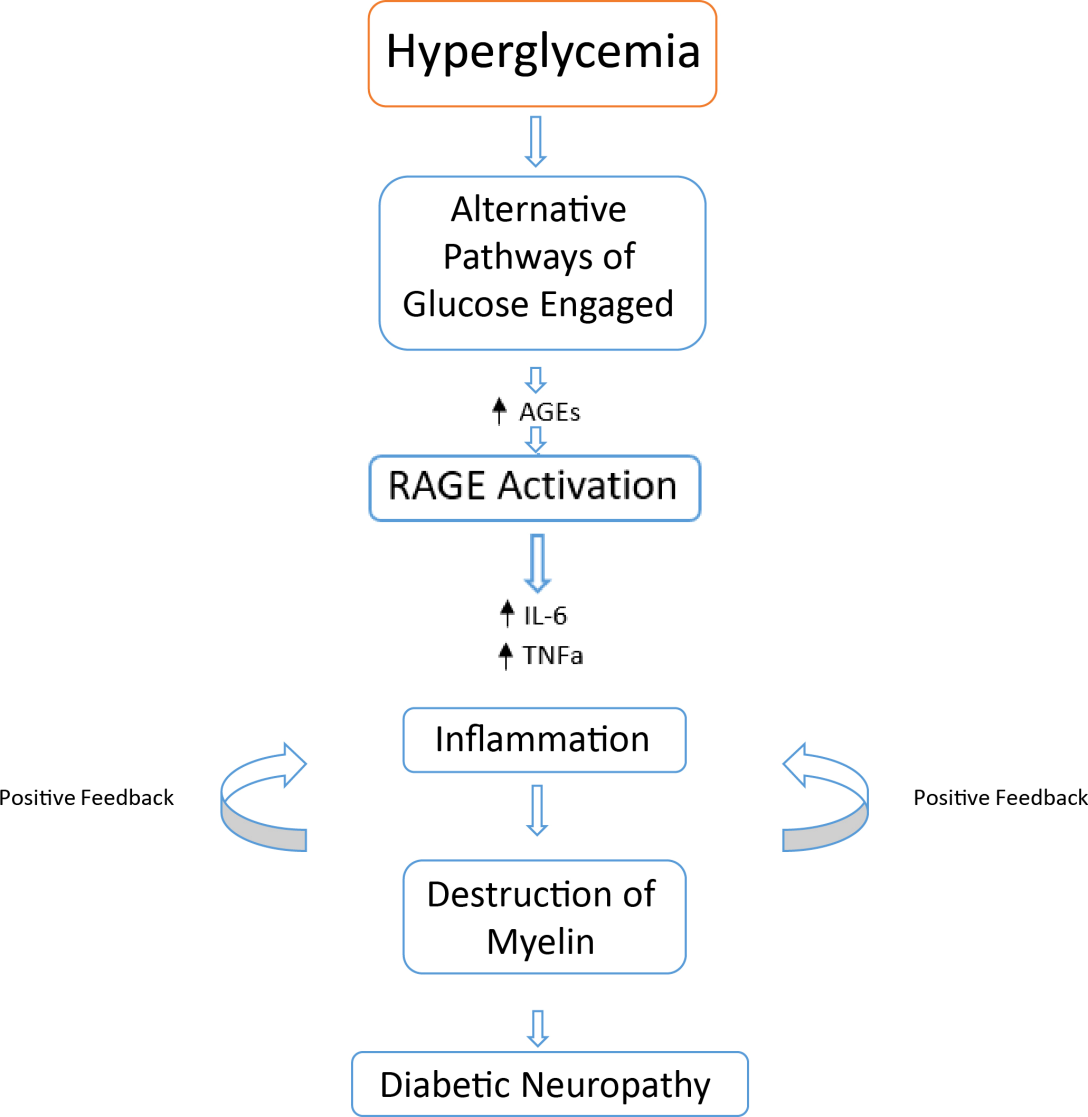
Basic overview of possible events leading to DPN. Chronic hyperglycemia alternatively activated glucose metabolism pathways result in increases of cytotoxic byproducts and end products. An increase in AGE - RAGE interaction results in an increase of proinflammatory cytokines leading to a positive feedback loop of immune cell activation. This leads to the destruction of nerve cell myelin, leading to a reduction in nerve functionality resulting in diabetic neuropathy.

Despite many clinical variations of diabetic neuropathy, the same root cause can be attributed to the disease progression. Hyperglycemia leading to the activation of alternative pathways of glucose metabolism (Polyol, PARP, protein kinase C, hedgehog and more) leads to the formation of AGEs, ROS, lipooxygenase pathways (LOX) and an elevation of proinflammatory cytokines (IL-6 and TNFɑ) (97) (**Figure 5**). The biochemical result of this, leads to cellular damage of the associated nerves as well as oxidative stress, altering the cellular and metabolic function in diabetic neuropathy (97). The principal proinflammatory cytokine involved in DPN is TNFɑ, as opposed to IL-6 as seen in other microvascular complications such as DR and DN (106–108). Activated macrophages, CD4^+^ cells, natural killer cells, mast cells and eosinophils upregulate TNFɑ to promote inflammation, as discussed earlier in DR and DN. Both diabetics and diabetics with neuropathy express more plasma TNFɑ compared to healthy controls (97, 109–111). Hyperglycemia overtime may cause nerve myelin protein glycosylation, resulting in possible recognition by specific macrophages at a later date, subjugating the myelin to phagocytosis (112, 113). Overtime, phagocytosis leads to demyelination of the nerve, causing suboptimal conduction of nerve signalling. When exposed, myelin antigens activate T cells which respond by secreting proinflammatory cytokines (namely TNFɑ) initiating an inflammatory immune response (112, 114). The cytotoxic effects of elevated levels of TNFɑ on oligodendrocytes may also cause further demyelination (115). The action of TNFɑ on mononucleated cells further amplifies the inflammatory response *via* secretion of IL-1β and IL-6.

Despite decades of research, progress in the medications combating diabetic neuropathy has not seen much progress, with clinicians primarily recommending higher standards of glycaemic control as well as exercise for prevention. There are no disease modifying therapies for those with DN, with most trials resulting in failure (116). As of the time of writing this review, most medication offered by clinicians is centred around the management of pain to improve quality of life. Anticonvulsants (e.g. pregabalin and lamotrigine), antidepressants (SNRIs and TCAs) and opioids (tramadol) are often used as therapies in diabetic neuropathy among others (100).

## Inflammation in diabetic patients

Common to all three (DR, DN and DPN) aforementioned pathologies is the notion of hyperglycemia being ultimately responsible for complications with diabetes. Correct diet, exercise and good glycaemic control are the ultimate end goals of patient care and can prove to be greatly preventative, however, this is ultimately an incredibly hard practise to maintain living with diabetes. Chronic hyperglycemia then, although ultimately responsible, will not be considered as a therapeutic target for this review, given the incredible number of factors that may or may not affect an individual’s blood glucose levels.

Alternative pathways of glucose metabolism (such as the polyol pathway) are the second step in the procession of diabetic complications, with hyperglycemia activation ultimately resulting in cytotoxic by-products. In tissues lacking sorbitol dehydrogenase, such as those associated with diabetic complications, research has been conducted in animal models and human cell lines (117, 118), however more research is needed into the effect on humans *in vivo*. These studies target the initial enzyme in the pathway, aldolase reductase, for inhibition with some success (119). Matoba et al. have reviewed the literature with regards to redox imbalances in DKD and illustrated that Sodium Glucose Transporter 2 inhibitors and Glucagon Like Peptide-1 receptor agonists are potential pharmaceutical treatments (120–123). A general lack of consensus on the effectiveness of aldolase reductase inhibitors therefore merits further research (14, 124, 125).

Although inflammation in the short term is beneficial for the body, chronic levels of inflammation can be very damaging, resulting in a myriad of complications (126). By targeting the inflammation common to most diabetic complications, one may lessen the burden or even prevent complications in the first place. Targeting the immune response provides many viable targets including; inhibition of soluble proinflammatory mediators, inhibition of transcriptional factors as well as inhibition of other immune processes (61).

Macrophages, T cells and the complement system all play an important role during inflammation with regards to diabetic complications. This inflammation in turn begets a vicious cycle of immune cell activation and further organ damage. As hyperglycemia induces alternative metabolic pathways of glucose, ROS and AGEs are produced with often problematic outcomes (127).

AGEs bind to corresponding AGE receptors (RAGE) causing upregulation of two transcription factors connected with inflammation, NF-κB and early growth response-1 (Egr-1) (128). In diabetic tissues and during inflammation, RAGE is correlated with the continuous activation of NF-κB, contributing to cell stress and dysfunction over a sustained period (128). This in turn triggers the adaptive immune system, in which RAGE has been evidenced to be upregulated during T cell activation in addition to monocyte chemoattractant peptide-1 (MCP-1) (128).

Macrophages are attributed as the principal inflammatory cell involved in kidney damage and are also known as the ‘remodelers’ (72, 129). This is owed to the presence of both proinflammatory type 1 (M1) macrophages stimulated by Th1 cells producing interferon gamma (IFN-ɣ), or the anti-inflammatory/repair mediating type 2 (M2) phenotype differentiated by Th2 cells secreting IL-4 and/or IL-13 (129, 130). Polarisation of macrophages is dynamic and may be altered depending on the cytokine inputs from the surrounding environment. When RAGE receptors are stimulated, the upregulation of MCP-1 causes monocytes to transform into macrophages ultimately resulting in local IL-6 and Tumour Necrosis Factor Alpha (TNFɑ) increases (129).

T cells also play a large role in inflammation. Although the recruitment process of T cells to the kidney in diabetes is poorly understood, it is known that they accompany the macrophages (61). The released IL-6 (from the previous paragraph) is known to activate Th17 cells in conjunction with TGF-β, and may very well be the driving factor of T cell movement to the diabetic kidney environment (131–133). Activated T cells secrete proinflammatory cytokines such as TNFα and IFN-ɣ. Both cytokines can directly damage kidney tissues through cytotoxic effects indirectly promoting the migration to (and activation of) macrophages in DN. Aforementioned AGEs may also bind to the RAGE on T cells, in turn stimulating T cells to secrete IFN-ɣ, leading to further kidney inflammation in what may be comparable to a positive feedback loop (61, 129).

In addition to macrophages and T cells, the complement system has been shown to play a role in diabetic complication development (69, 134, 135). The complement system pertains to the numerous small proteins produced by the liver aiding in the clearance of pathogens and damaged cells from the body. However during hyperglycemia, the glycation of these complement proteins leads to dysregulation of the complement pathways as well as pattern recognition molecules of the complement system binding to glycated ficolins activating the leptin complement pathway in DN (134). Activation of the complement system in turn promotes inflammation ultimately causing further damage in diabetes. Clinical trials acknowledging the role of complement in DN development have targeted elements of the pathway for deletion, resulting in better disease outcomes (discussed later in the review).

Inflammation from macrophages and T cells may be hampered by the presence of regulatory T cells (Tregs) (136) among other methods. This review will now discuss the various possible methods of lessening inflammation in the aforementioned diabetic complications.

## Therapeutic strategies

There are a number of proposed means to prevent inflammation culminating in diabetic complications. The following section includes several of these potential therapies to decrease the effect of inflammation.

### Regulatory T cells

Tregs, (CD4^+^CD25^+^CD127^-/low^Foxp3^+^) are part of the adaptive immune system that can decrease the immune response in order to maintain immune homeostasis. Initially, Tregs were thought to be a terminal phenotype, however, evidence has emerged of their capability in plasticity, as well as their dynamism (84, 88, 137, 138). In autoimmune patients, such as diabetics, the effectiveness of Tregs are often diminished due to a variety of possible reasons including; poor stability of Foxp3 expression (84, 139–141) or production of proinflammatory cytokine IFN-γ in aberrant processes (84, 88, 137, 142, 143). Our group has also reported that Treg cells switch their phenotype in T1D under lack of IL-35 (142–144).

Although it is possible to generate Tregs through the manipulation of cytokines and co-stimulation, the remaining issue is the functional efficiency of Tregs. Transcription factor Foxp3, a defining characteristic of a Treg cell, must be stable in order to function as a Treg cell (87). *In vitro* studies have documented that exposure to proinflammatory cytokines lead to loss of Treg cell stability, producing an “exTreg cell” (145). This same phenomenon is observed *in vivo* when Treg cells are transplanted into autoimmune mouse models, where the exTreg cells then become memory T cells and/or effector T cells (140, 146). Komatsu et al., investigating the plasticity of Treg cell populations, discovered that CD25^+^ cell populations exhibited a more stable lineage of Treg cells as opposed to CD25^-^ (86). They had found that Foxp3 expression alone would not sustain a population of Tregs, finding that some Tregs (CD25^-^) would alter phenotype to Th17 T helper cells, confirmed by other studies (147, 148).

Approximately 0.5% of B cells belong to a subset known as regulatory B cells (Bregs) (149). Although the extracellular markers for this population are still being studied, what is known is that they release cytokines including IL-10, IL-35 and Transforming Growth Factor beta (TGF β) to transform Th1 and Th17 T helper cells into Treg cells (149–151). It has been shown in chimeric mice that by lowering IL-10 producing B cell numbers, one can lower Treg cell numbers and thus increase the amount of Th1 and Th17 T helper cells (142, 152). Hence by providing cytokines which encourage Breg cell differentiation, one would expect an increase in Tregs and thereafter a decrease in inflammation.

### IL-35

IL-35 is an anti-inflammatory heterodimer cytokine from the IL-12 family composed of IL-12α (p35) and Epstein-Barr virus-induced gene 3 (Ebi3) protein chains which play a key role in the suppressive function of Tregs (153). IL-35 is secreted by a number of immune cells *in vivo* including both Treg and B regulatory cells as well as tolerogenic dendritic cells and pro metastatic cancer cells (153, 154). On binding with a corresponding cytokine receptor, IL-35 activates the Janus Kinase-signal transducer and activator of transcription pathway (JAK-STAT). The exact molecular pathways and function of IL-35 binding still remains to be totally illustrated in inflammatory autoimmune diseases (155). IL-35 is associated with many autoimmune conditions including: T1D, rheumatoid arthritis, multiple sclerosis, systemic lupus erythematosus, primary Sjögren syndrome and atherosclerosis in addition to cancer metastases (153). What is known however, is that IL-35 has been reported to have an anti-inflammatory effect by expanding Treg cell populations and preventing development of proinflammatory Th1 and Th17 cells (156).

Our group has found that administration of IL-35 reversed established T1D in NOD and MLD-STZ mice by maintaining the suppressive phenotype of Treg cells (143). In addition, we found that levels of IL-35 were lower in patients with recent onset and long standing T1D than in healthy controls. A decrease in IL-35 positive Treg and Breg cells has been reported by our group in patients with T1D compared to healthy controls (157). With similar results to our earlier studies, we also found that IL-35 maintains the suppressive phenotype of Breg cells in experimental T1D.

In a study performed by Jiang et al., the STZ rat model was used to simulate DN (158). In this study, Jiang et al. found that by administering IL-35 the progression of inflammation was prevented by the reduction of proinflammatory cytokines IL-1β, IL-6 and TNFɑ and by increasing IL-10 (an anti-inflammatory cytokine). Yan et al., using an STZ mouse model for diabetic retinopathy, also found very similar results in line with Jiang et al. (159). Patients with DR were found to have significant downregulation of IL-35 in vitreous tissues compared to controls (159). IL-35 administration had a suppressive effect on Th17 T helper cells, ultimately causing less inflammation and thus providing more evidence of the therapeutic effect of IL-35 in preventing experimental DR (160).

IL-35 treatment was also found to polarise classically activated proinflammatory M1 macrophages to an alternatively activated anti-inflammatory M2 phenotype (161). M1 macrophages, when stimulated by TNFɑ secreted by Th1 cells, release TNFɑ, IL-1β and ROS as part of a positive feedback mechanism of inflammation (162). A recent study by Luo et al. found that IL-35 treatment prevented the elevation of M1/M2 ratio in MLD-STZ mice, a model used for experimental T1D. In addition to this, the proportion of TNFɑ^+^ cells among macrophages in PDLN was decreased, with unaffected TNFɑ^+^ cell numbers in the spleen, maybe suggesting that effects are localised as opposed to systematic (162).

As observed in the aforementioned studies, the role of IL-35 in maintaining a population shift toward Foxp3 expressing Tregs and away from Th17 T cells was important, thereby reducing the amount of inflammation at target sites. IL-35 also holds the ability to shift proinflammatory M1 macrophages to anti-inflammatory M2 macrophages, reducing the secretion of TNFɑ among M1 macrophages. The value of IL-35 in patients with diabetic complications is of great importance and is therefore a hopeful choice for the future treatment of diabetes and its subsequent complications after safety testing in humans. As well as the use of cytokines, other techniques may be utilised to increase Treg quality and therefore lower inflammation.

### CRISPR editing of Tregs for higher stability

An innovative method used to edit the genome in cells, CRISPR/Cas9 (clustered, regularly interspaced, short palindromic repeats/CRISPR associated protein 9) technology is being investigated to expand viable Treg numbers (163–166). By using CRISPR, one could target either disabling or enhancing pathways of Treg cell functionality.

One such pathway leading to the instability of Treg cells, moderated by IL-6, has been well documented and features in mAB treatment for autoimmune and inflammatory conditions such as SLE and rheumatoid arthritis (163, 167, 168). Genes associated with the IL-6 alpha (IL-6α) receptor (CD126) were knocked out in a study by Zeebroecket al. (163). The resulting Treg cells showed stable expression of Foxp3 and Helios, IL-2, IL-10, IFNγ and IL-17a compared to mock Tregs whilst showing significantly lower phosphorylation of STAT3 (a pathway activated by IL-6). By using CRISPR to target and delete the IL-6α gene, the ratio of Th17/Treg cell may shift in favour of Treg cells.

Hypoxia-inducible factor 1-alpha (HIF‐1α) is a subunit of HIF-1 encoded by the HIF-1α gene (169). Primarily, HIF-1α is activated in hypoxic conditions to upregulate genes associated with angiogenesis, erythropoiesis and glycolysis (170–175), however it is also induced by continuous TCR stimulation *via* mTOR in human T cells (165). In mice and human embryonic kidney cells, HIF-1α is capable of downregulating Foxp3 expression either by ubiquitination or proteasomal degradation even in non-hypoxic conditions (176). In HIF-1α deficient mouse models, the balance of Th17/Treg cells is disrupted to favour Treg cells (176).

More and more research is being conducted on potential genes involved in Foxp3 stability (177). As more gene functions are discovered, more targets emerge for CRISPR intervention leading to potential trials *in vitro* and *in vivo*. By increasing the stability of Tregs *via* CRISPR, the potential to effectively reduce the damage caused by inflammation also increases. This gives us a potential to treat or prevent diabetic complications, should trials be successful. In addition to the editing of Tregs through CRISPR, it is also possible to produce Tregs through stem cell technologies.

### The effect of mesenchymal stromal cells on Tregs

NEPHSTROM is a newly proposed therapy funded by EU Horizon showing great promise for DKD treatment as well as a treatment for other micro- and macrovascular complications (96, 178, 179). Versatility is an attractive feature of mesenchymal stromal cells (MSCs), allowing them to differentiate into a number of cell types. This allows them to initiate tissue repair, restore lost function, migrate to sites of injury and target effector mechanisms associated with inflammation (180). *Ex vivo*-expanded MSCs and T-regs have been used successfully, and most importantly safely, in many animal models due to their immunomodulatory effects. This makes them an extremely attractive option in the treatment of autoimmune disease (180). The preclinical ORBCEL-M data provided a basis to further investigate the role of MSCs in slowing or stopping progressive DKD (181).

The specific mechanism of action is still to be clarified; however, it is commonly held that cell-to-cell contact dependent mechanisms alongside co-stimulation and soluble mediators play an important role (180). High affinity binding of MSCs with T-cells *via* upregulated expression of cell integrins, ICAM-1, VCAM-1, CD71 and CD58 play an important role in immune suppression. Notch receptors, expressed highly on MSCs, also stimulate iTreg cell differentiation, which better resist IL-6 conversion to Th17 phenotypes in inflammatory conditions compared to pTregs (182). Interaction with antigen presenting cells such as dendritic cells has also been identified as a mechanism of action of MSC therapy. Bone marrow-derived MSCs can reprogram mature dendritic cells into antigen presenting cells with distinct jagged-2-dependent regulatory properties. This in turn causes CD4^+^ T cells to differentiate into Tregs effectively repressing Th17 differentiation (183, 184).

The regenerative potential of MSCs can depend on other factors, since several exogenous (and other) factors may greatly impact the MSC’s biological properties and subsequent function (185, 186). Although the majority of MSC based therapy remains promising, the potential of some adverse effects is not zero, with some researchers highlighting that the effects of paracrine signalling and the secreteome have yet to be fully examined (185, 187).

### Inhibitors of the complement system

As stated in the introduction, the complement system has also been identified to play a key role in diabetes related inflammation, particularly in cases of DKD (61, 134). Research in this area is still relatively new however, with only a small number of studies conducted on mice (188) and rats (189). In a study by Li et al, C3a was identified as a viable drug target in DN after rats were given a high-fat diet + repeated low dose STZ to induce diabetes in addition to a C3aRA versus a control group. After 8 weeks, biochemical analysis showed that mice treated with C3aRA had an improved renal function and morphology, compared to diabetic rat controls. Thus the complement system is a valid therapeutic target in DN.

Eculizumab is a humanised monoclonal antibody which binds to C5, blocking its ability to form the membrane attack complex and is already in clinical use in other diseases (61, 190). Although the question of whether these drugs will have any effect on the microvascular complications of diabetes remains to be answered (135).

The key issues remaining for complement inhibition pertains to the localisation of the effect to a target organ as well as dosing. Systemic effects of complement inhibition render the patient susceptible to infection, whilst the abundance and high turnover of complement proteins in the blood plasma present a challenge to dosing (191). Thrombomodulin has also been suggested as a target, as it is a regulator of both complement and coagulation activation as diabetic mice lacking thrombomodulin lectin-like domain had exasperated DN (135, 188).

In addition to the Tregs and the complement system, macrophages may also be targeted in order to alleviate the burden of inflammation on organs associated with diabetic complications.

### Transcription factor EB

MSCs also have the ability to convert M1 macrophages to M2 macrophages *via* utilisation of Transcription Factor EB (TFEB). Classical M1 activation of macrophages begins with TLR activation on the macrophage surface alongside INFɣ stimulation. The activated M1 macrophage secretes proinflammatory cytokines, reactive nitrogen/oxygen species and promotes the Th1 response among other attributes (192). Alternative macrophage activation (M2) occurs in response to stimulation from IL-4/IL-13 and is known to induce tissue remodelling responses (192). M2 macrophages also reduce expression of MHC-II and co-stimulatory molecule CD80, consequentially reducing the activation of the T cell response.

These phenotypes are not terminal and may shift *in vitro* and *in vivo.* Although the exact mechanism responsible for M1 to M2 polarisation is still unclear, a study conducted by Fang et al. documented the effect of TFEB on macrophage polarisation in cancer. They found a reduction in TFEB promoted M2 polarisation *via* reduced SOCS3 expression and enhanced STAT3 signalling; whilst chemical over expression mediated *via* hydroxypropyl-β-cyclodextrin resulted in M2 macrophage polarisation inhibition (193). Tumours, when secreting TGF-β ultimately begin a cascade of intracellular reactions leading to M2 macrophage polarisation and a reduction in T cell response. Notably TGF-β is also secreted by Treg cells (194).

The therapeutic effect of TFEB has already been observed in conjunction with chemotherapy, where the presence of TFEB has increased metabolic pressure and therefore increased tumour sensitivity to chemotherapy treatment (195). The effect of TFEB is unclear in diabetic conditions, however an investigation by Song et al. showed that in diabetic mice, levels of activated TFEB (p-TFEB S142) increased in aortic endothelial cells whereas the total protein count of TFEB decreased (196). They found that diabetic factors such as IL-1β and insulin suppressed TFEB and suggest TFEB as a means of treating inflammation associated with diabetes, particularly aortic inflammation. By utilising RNA-seq, they identified key proinflammatory pathways regulated by TFEB, most importantly the effect TFEB has on NF-κB inhibition thereby suppressing the expression of proinflammatory genes such as VCAM1, ICAM1, SELE, and CCL2.

### TNFɑ inhibition

At the time of writing, approximately five approved TNFα inhibitors exist on the market for treating inflammatory conditions such as rheumatoid arthritis, crohn’s and psoriasis as well as some off label alternatives (197, 198). Although largely successful as a therapeutic measure, there have been documented cases of adverse events, such as hypersensitivity reactions (46, 199). Further analysis conducted by post-market surveillance and clinical trials have detailed that some patients also run the risk of developing infections, autoantibodies, malignancies, paradoxical inflammation and demyelinating disorders (197, 199, 200).

A case study of a 35 year old woman who had been suffering from rheumatoid arthritis since the age of 15 was documented by Tack et al. (198). After beginning an anti-TNFα treatment (etanercept; dose 25mg 2x per week) in late 2000 she saw a beneficial effect, where her arthritis was reported to be in a much better condition using appropriate metric tests, however her joint inflammation persisted. At this juncture, her fasted blood sugars were within the homeostatic range of 5.7 and 6.1 mmol/l. The patient’s etanercept therapy continued, however, in 2003 the patient developed T1D. Although onset of the disease was not attributed to the treatment by the authors, it was also not ruled out. It was observed that anti-TNFα treatment did not prevent the development of diabetes. There has also been a further example of an arthritis sufferer developing T1D after beginning anti-TNFα therapy (201).

Anti-TNFα therapy on those already diagnosed with diabetes has seen mixed results. In one case study, a T1D patient receiving anti-TNFα treatment (etanercept) for arthritis lost stable control of her blood glucose levels, where they were described as “erratic” and lead to severe hypoglycemic attacks without warning, having previously had stable control (202). When the treatment was discontinued, her blood glucose control had returned to a stable condition. This was attributed to TNFα’s ability to cause insulin resistance in adipose tissue by down regulating glucose transporter mechanisms (203).

Treatment of anti-TNFα may also lead to demyelination of the central nervous system. A review conducted by Fromont et al. documented two patients who showed MS-like symptoms after beginning anti-TNFα therapy and a further who was diagnosed with MS (114). In a review of the post-marketing database of the US Food and Drug Administration, Shin et al. found a total of 15 patients were observed to develop Guillain-Barré syndrome (or variants of the syndrome) after taking different versions of anti-TNFα medication (204). It is therefore recommended that those with an underlying demyelinating disease (potentially such as those with a diabetic neuropathy) do not take anti-TNFα therapy (197).

Although anti-TNFα treatment may work for some, the risk of developing a complication is not insignificant. Until more research is conducted on the safety of other treatments, it is hard to comment on the effectiveness of TNFα as a target in diabetic complications. It is not evidently clear if diabetic neuropathy is a demyelination or axonal disease, or subsetted into either (205, 206), and therefore the treatment of diabetic neuropathy using anti-TNFα may potentially cause damage.

### IL-6 inhibitors

IL-6, although proinflammatory, is also very important in other homeostatic functions. It is involved in the insulin-induced clearance of glucose in the liver and skeletal muscle, released by skeletal muscle during exercise and alters insulin secretion by increasing the secretion of glucagon-like peptide 1 (207). The duality of both the pro and anti-inflammatory potential of IL-6 is thought to be based on which pathway is involved in its activation. For example, IL-6 proinflammatory signals (notably in DR) are caused by trans-signalling whilst regenerative or anti-inflammatory activities of IL-6 are thought to be caused by classical activation (208).

Anti-IL-6 (sarilumab) has been seen to lower HbA1c in phase III clinical trials when used as a monotherapy (209). However, in an interesting contrast, a paper by Jörns et al. observed that anti-IL6 alone was insufficient at sustaining an anti-diabetic metabolic state. By studying the effect of various combinations of either anti-TCR, anti-IL-6, anti-IL17A treatments on LEW.1AR1-iddm (IDDM) rats, it was established that normo-glycemia was restored best in combination of all three treatments (210). Although appealing as a preventative measure of diabetic complications, anti-IL-6 treatment may also hold potential in related disorders or complications (207). However much more research into the anti-IL-6 is needed and if appropriate, which combination works most favourably at restoring blood glucose levels.

### IL-17a inhibitors

IL-17a is an important inflammatory mediator in chronic inflammation, especially in examples of kidney disease and diabetic complications (211). As such, IL-17 inhibition has been a target for some clinical trials in other diseases relevant to chronic inflammation such as rheumatoid arthritis and psoriasis. Although well tolerated with good safety profiles, there have been some observations made of adverse events in IL-17 and IL-23 inhibitors compared to placebo controls (212). These include infections, nasopharyngitis and headaches as well as local injection site reactions in a trial for ixekizumab (an IL-17 inhibitor).

In their 2019 review of IL-17a as a novel target, Lavoz et al. considered ongoing clinical trials of anti-IL-17a drugs as well as FDA and EMA approved medication (for patients of psoriasis and arthritis) and generally found them to be safe and well tolerated (211, 213, 214). They further comment on the effectiveness of IL-17a inhibitors over TNFɑ inhibitors in the same conditions and ultimately suggested that IL-17a inhibitors be considered for investigation in diabetic complications.

### Ceramide treatments

Ceramides, a molecule of the sphingolipid family, are crucial for the stability of mammalian cell membranes and are gaining more attention for their role in inflammation (215). Their metabolites sphingosine-1-phosphate (S1P), and ceramide-1-phosphate (C1P) are involved in a variety of cell survival traits, including growth and immune cell trafficking as well as inflammation (216, 217). The rate of ceramide generation is dependent on the availability of long-chain saturated fatty acids (218). Ceramide is generally proinflammatory, however S1P and C1P can be either proinflammatory or anti-inflammatory depending on the cell type (219).

Ceramides are implicit in T2D progression with some groups suggesting its use as a biomarker (220). They have been observed in high levels in tissues relevant to diabetic micro- and macrovascular complications (220). The novel use of anti-ceramide treatment could lead to a therapeutic effect in various diabetic complications. The effect of anti-ceramide scFv immunotherapy in retinopathy was recently investigated and found that one injection of scFv at hyperglycemia onset prevented an increase in retinal vascular permeability (221). Although the sample size was relatively low, the results suggest that scFv immunotherapy may be beneficial in early stages of diabetic retinopathy, however, further research would of course be necessary.

### Klotho

Klotho is a transmembrane protein expressed predominantly in the kidneys and parts of the brain as well as other regions of the body in lower concentrations (222). The ecto domain, soluble klotho (sKL), acts as a hormone, activating many molecular pathways (223). It is often associated with anti-aging and has noted beneficial systemic effects, namely suppression of nuclear factor NF-κB activation and increasing lifespan (224). In db/db mice, renal Klotho (mRNA and protein) is significantly lower, further suggesting the role of Klotho as an agent of anti-inflammation (225). This is also true with regards to serum levels of soluble Klotho during T1D onset, where children have significantly lower levels compared to healthy controls (226). Furthermore, Klotho deficiency promotes β-cell apoptosis in T1D, where over-expression in turn improved β-cell function and offered a protective effect (224). By reducing the effect of the NF-κB pathway, adhesion molecules ICAM-1 and VCAM-1 are suppressed, preventing endothelial dysfunction.

The use of sKL for its anti-fibrotic effect in the kidney is of particular interest to the discussion of novel targets for DN. One mechanism suggested for nephroprotection is *via* the blockade of profibrotic TGFβ1 signalling (223). The problem with sKL however, is its relatively short half-life and large size, which is hard to reproduce in bioactive form. In their 2022 review, Isakova, Yanucil and Faul discussed research on one of two peptide domains on sKL, namely KL1 (223, 227). Although its role is largely unknown, it is thought to be involved in TGFβ1 signalling. It was further discussed in the same review that KP1 binds to TGFβ receptor 2, thus disrupting normal functioning of TGFβ signalling such as myofibroblast activation. *In vivo* studies show that KL1 has preference for accumulation in the kidney cells after intravenous injection and has antifibrotic properties (227).

With a length of 30 amino acids, KL1 is much smaller than sKL, and therefore easier and cheaper to replicate. However, a large volume of studies must still be conducted to assess various aspects of safety. Klotho appears to answer many of the issues seen in diabetic complications and onset, however much still remains to be learned regarding its potential as a therapeutic. Its promise as a protein that can offer protective effects in both early onset of diabetes as well as the subsequent complications cannot be understated.

### DNAse I

In 1997, a landmark paper by Takei et al. documented the neutrophil’s ability to dispense decondensed chromatin into the extracellular space to form a neutrophil extracellular trap (NET), later interpreted and termed by Brinkman et al. (228, 229). Subsequent research has recorded disruptive behaviour as a result of NET formation *in vivo* such as clot formation as well as sterile inflammation in cancer (230, 231). NET formation has also been observed in diabetes and is thought to be involved in many complications from diabetes (230, 232, 233). Neutrophils follow chemokine gradients in order to find pathogens or damaged tissues and often, when forming NETs, release more proinflammatory cytokines (234). Although the exact mechanism behind NETosis is still unclear, NETs are known to form in hyper glycemic environments with lower pH and high ROS (40, 234).

Deoxyribose nuclease I (DNase I) is an *in vivo* serum endonuclease excreted by non hematopoietic cells. Among other purposes, DNase I acts to remove NETs by targeting the phosphodiester bonds of the exposed DNA. A study by Jiménez-Alcázar et al. showed that *in vivo*, two forms of DNase I (DNase I and DNase 1 like 3) work to degrade NETs in circulation (235). A genetically engineered form of recombinant human DNase I (called Dornase Alfa) is already used for human patients with cystic fibrosis (236, 237). After inhaling recombinant human DNase I (rhDNase), patients showed an increase in their ability to clear airway obstructions compared to healthy control groups (238). RhDNA is safe, with no immune reactions, local irritation or major adverse events. A study conducted by Davis et al. evaluated an IV injection of rhDNase in patients with SLE. They found no immune response or other adverse effects pre or post treatment to rhDNase following IV administration. It was further observed by the group that serum rhDNase concentrations were still sufficient for the hydrolysis of NETs after a few hours post administration (239).

More research into the effects of NETosis with regards to diabetic complications provides interesting results. Circulating biomarkers for Netosis include circulating DNA–histone complex and polymorphonuclear neutrophil elastase levels are elevated in diabetics with DR compared to diabetics without DR (240). A multivariable logistic regression model, accounting for fasting blood glucose and HbA1c levels, highlighted that NET formation biomarkers are significant independent risk factors for those with DR (240, 241).

These studies among others are beginning to highlight the importance of considering the role of NETs in diabetic complications. By developing further forms of DNase I with an increased half-life, such as the IV study conducted by Davis et al., investigations into the effects of NET formation on diabetic complications could provide insight into novel target therapies.

## Conclusion

As the global incidence of diabetes is increasing, more people are proportionally likely to develop diabetic complications as a result. Current medication mainly provides pain relief in order to better the patient’s quality of life. With increasing evidence validating chronic inflammation and the immune system’s impact on the development of diabetic complications, immunotherapy may play a vital role in the development of new medications for such conditions.

The suggested therapies of increasing Treg cell numbers *via* IL-35, CRISPR, MSCs or administration of their secretions (TGF-β) has been shown in diabetic animal models to restore immune homeostasis and return glycemic values to appropriate levels. By targeting other pro-inflammation mediators such as IL-6 or IL-17A one may further support the development of Treg cells. Other anti-inflammation therapies such as anti-ceramide treatment or klotho may take some more time for development, but hold great potential as novel targets. More investigations into DNase I in diabetics may prevent NET formation and have a beneficial effect overall.

Although prevention is the best means to avoid diabetic complications, great progress has been made by research teams in investigating novel targets of the immune system with the aim to prevent disease development. Consideration of the above therapies with a good glycemic control could potentially improve the quality of life for those living with diabetes.

## Author contributions

Conception: KS; research: LR, KS and ZL; LR and KS wrote the manuscript. KS arranged research fundings and supervised the study, which was revised by ZL; diagrams: LR. All authors contributed to the article and approved the submitted version.
